# Population-based screening of newborns: Findings from the newborn screening expansion study (part two)

**DOI:** 10.3389/fgene.2022.867354

**Published:** 2022-09-01

**Authors:** Kee Chan, Amy Brower, Marc S. Williams

**Affiliations:** ^1^ American College of Medical Genetics and Genomics, Bethesda, MD, United States; ^2^ Geisinger Health System, Danville, PA, United States

**Keywords:** newborn screening, genomics, pilot studies, metabolic disease, immunodeficiencies, ducheme muscular dystrophy, public health, population based screening

## Abstract

Rapid advances in genomic technologies to screen, diagnose, and treat newborns will significantly increase the number of conditions in newborn screening (NBS). We previously identified four factors that delay and/or complicate NBS expansion: 1) variability in screening panels persists; 2) the short duration of pilots limits information about interventions and health outcomes; 3) recent recommended uniform screening panel (RUSP) additions are expanding the definition of NBS; and 4) the RUSP nomination and evidence review process has capacity constraints. In this paper, we developed a use case for each factor and suggested how model(s) could be used to evaluate changes and improvements. The literature on models was reviewed from a range of disciplines including system sciences, management, artificial intelligence, and machine learning. The results from our analysis highlighted that there is at least one model which could be applied to each of the four factors that has delayed and/or complicate NBS expansion. In conclusion, our paper supports the use of modeling to address the four challenges in the expansion of NBS.

## Introduction

In the United States, every year, at least 12,905 babies are identified with genetic disease by population-based newborn screening (NBS) (Sontag et al., 2020). The goal of NBS is to enable the early diagnosis and treatment of disease in newborns to improve health outcomes, at both an individual and population level. While screening is directed at newborns, the health benefits of a positive screen can be multiplied through the testing of parents, siblings, and other at-risk relatives (known as cascade testing), and this increases the population impact of NBS screening (Caggana et al., 2013). NBS is a complex but well-established system involving diverse stakeholders, including researchers, state public health departments, pediatricians and family physicians, subspecialists and geneticists, industry, parents and advocates, and federal agencies. These entities contribute to the key components of NBS: 1) Prenatal Education, 2) Laboratory and Hospital-based Screening, 3) Diagnosis, and 4) Medical Management/Surveillance (www.aphl.org).

NBS began in the 1960s with screening for a single disorder. It has expanded over time and now encompasses a recommended uniform screening panel (RUSP) of 35 core and 25 secondary conditions^1^ that the Secretary of the Department of Health and Human Services (HHS) recommends for states to screen as part of their state NBS programs and up to an additional 20 non-RUSP conditions screened in at least one state (https://www.hrsa.gov/advisory-committees/heritable-disorders/index.html; www.newsteps.org; and www.nbstrn.org)^2^. On the Newborn Screening Translational Research Network (NBSTRN) website (www.nbstrn.org), information regarding the composition of state NBS panels, including the RUSP and non-RUSP conditions can be found on the NBS-Virtual Repository of States, Subjects & Samples (NBS-VR) data tool, which provides national and state-level views of these policies and procedures, and the NBS Conditions Resource (NBS-CR), which provides a centralized resource^3^ of facts and statistics for each condition. The expansion of NBS increases the number of screened conditions and is usually triggered by the approval of novel therapies and interventions, or the discovery of new screening or diagnostic technologies (McCandless and Wright 2020). With rapid advancements in genomic technologies to screen, diagnose, and treat newborns, there are conceivably hundreds to thousands of conditions that could be detected; however, not all would be considered as candidates for NBS and for NBS pilots (Berg et al., 2017; Milko et al., 2019). Historically, the evolution of a condition from being a candidate for NBS to implementation of nationwide screening involves a series of steps and pilots conducted by researchers and state NBS programs that are supported by advocacy groups, industry, and/or federal agencies (such as National Institutes of Health (NIH), Center for Disease and Control Prevention (CDC), and Health Resources and Services Administration (HRSA)).

**FIGURE 1:**
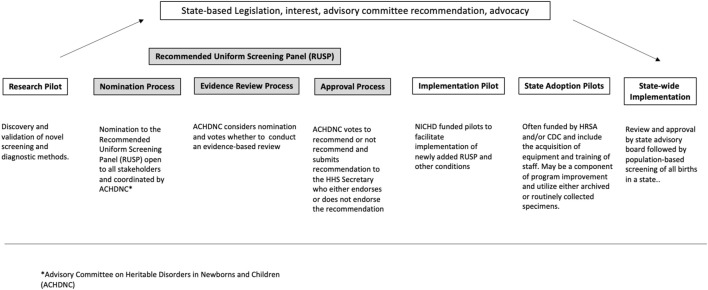
Pathway of candidate conditions (adapted from Brower et al., 2022).

To review the expansion of NBS and the role of NBS pilots in this expansion, NBSTRN conducted the NBS Expansion Study which included a meeting of experts and a series of analyses summarized in our companion paper “*Population-based Screening of Newborns: Findings from the NBS Expansion Study* (Part One)” (Brower et al., 2022). NBSTRN is a resource for investigators engaged in NBS-related research led by the American College of Medical Genetics and Genomics (ACMG) and is funded by a contract from the *Eunice Kennedy Shriver* National Institute of Child Health and Development (NICHD). Brower et al. describes the current approach to expansion that uses research and implementation pilots of short duration and limited sizes in a small number of states, followed by condition-by-condition review by a federal advisory committee, and state by state adoption (Figure 1, adapted from Brower et al., 2022). Brower and others found that the current system of NBS expansion is not able to keep pace with the pipeline of NBS screening and pilot candidate conditions and described in detail four factors that delay and/or complicate NBS expansion (Table 1). In this paper, we describe how decision modelling can be used to address these four factors in a cost-effective and efficient way. This purpose of the paper is a call to action for additional resources to support research in developing, hypothesis testing, and applying of the use of models in NBS pilot studies.

**TABLE 1:** Four challenges in newborn screening pilot*.

Factor 1. Variability in screening panels persists.
Factor 2. The short duration of pilots limits information about interventions and health outcomes.
Factor 3. Recent RUSP additions are expanding the definition of NBS.
Factor 4. The RUSP nomination and evidence review process has capacity constraints.

* These four challenges are discussed further in Brower et al (2022)

## Suggestions on the use of models to facilitate the expansion strategies in newborn screening

The fundamental purpose of decision modeling in public health is to compare different policy options or strategies by calculating and comparing the expected value of the outcomes that result from the possible choices. Models have been used to simulate clinical trials, hypothetical scenarios, and projection of cost-effectiveness analysis (Prosser et al., 2013). In the context of NBS expansion, decision analysis that uses models can provide a quantitative analysis of all the relevant inputs (e.g., resources, screening parameters, incidence, cost of treatments) according to their probabilities (e.g., disease prevalence, likelihood of disease onset) and their relative importance at the different stages of NBS expansion from research pilots to nationwide implementation (Figure 1). Research studies are mechanisms to discover novel technologies to screen, diagnose, treat, and manage NBS conditions, and clinical trials are conducted to establish the safety and efficacy of treatments. Both efforts inform NBS pilot studies that most often assess the analytical and clinical validity of screening and, in some cases, diagnostic methods. Taken together, research studies, clinical trials, and NBS pilots by their design generate only a small fraction of the knowledge needed to inform the broad clinical implementation and public health practice changes are needed to realize NBS expansion. Decision modelling could be used to address these limitations and augment the information derived from research, clinical trials, and NBS pilots, ultimately improving the current approach to NBS expansion (Caggana et al., 2013; Gantt et al., 2016). This type of modeling may be helpful for the several scenarios encountered in NBS expansion and rare diseases including: different population sizes, the often very low disease incidences of rare diseases that are candidates for NBS and NBS pilots, variable costs of and access to treatments and interventions, and differences in analytical approaches based on individual state practices, including screening algorithms and thresholds for determining screen positives, access to expertise, and resources for follow-up and treatment.

## How do you select the models to use?

To identify model(s) that could address the four factors in NBS expansion, we reviewed the literature to find models that have been used to address similar problems. In addition to our literature review, we noted that models have been and are being used in NBS. Examples include the decision analytic modeling that is currently conducted during the evidence-based review of conditions considered by the Advisory Committee on Heritable Disorders in Newborns and Children (ACHDNC). Over 100 articles were reviewed and we identified models that were applicable to support the development, implementation, and expansion of NBS for rare diseases (Boshuizen et al., 2001; Carroll and Downs, 2006; Castilla-Rodríguez et al., 2017; Chan et al., 2011; Ding et al., 2016; Gantt et al., 2016; Hamers and Rumeau-Pichon, 2012; Kemper and Downs, 2000; Khneisser et al., 2015; Pandor et al., 2004; Peterson et al., 2013; Thiboonboon et al., 2015; Tran et al., 2007; van den Akker-van Marle et al., 2006). In our inclusion criteria, we reviewed models that were used and could be used for the following purposes: 1) research studies including efforts to discover and validate new technologies and treatments, 2) research pilots, 3) implementation pilots, and 4) state adoption pilots in newborn screening. Because the NBS system includes public health and clinical care, we also searched for models used to address similar system level challenges encountered in healthcare. These models may be particularly helpful in NBS expansion if they prove useful and future efforts are given the opportunity and support to further explore their value. To expand the application of other models to NBS expansion and pilot studies, we also included literature from the fields of system sciences, business, economics, and healthcare. From this review, we summarized the commonly used decision analytic models that may be appropriate for NBS decision-making (Table 2). It is important to note that this list is not exhaustive; rather, this list should act as an open invitation for all NBS stakeholders to explore and apply models to address NBS expansion challenges (Table 3).

**TABLE 2:** Types of analysis derived from modelling.

Type of analysis	Description	References
Economic evaluation	A process of systematic identification, measurement and valuation of the inputs and outcomes of two alternative activities, and the subsequent comparative analysis of these	Grosse et al. (2016); Prosser et al. (2013); Wright et al. (2015)
Programmatic cost analysis	A process to compare the program costs to program outcomes which can include all the resources required to implement an intervention, including personnel, space and utilities, travel, materials, and supplies	Bessey et al. (2018)
Cost-effectiveness analysis	A process that examines both the costs and health outcomes of one or more interventions and compares an intervention to another intervention (or the status quo) by estimating how much it costs to gain a unit of a health outcome, such as a life year gained, or a death prevented	Castilla-Rodríguez et al. (2017); Chan et al. (2011); Kemper and Downs, (2000)
Cost of illness analysis	A method of measuring medical and other costs resulting from a specific disease or condition	Tran et al. (2007)
Cost-benefit analysis	A systematic approach where the program costs and benefits are converted into dollars to estimate the strengths and weaknesses of alternatives used to determine options which provide the best approach to achieving benefits while preserving savings	Ding et al. (2016); Khneisser et al. (2015); Lord et al. (1999)
Cost-utility analysis	A special type of cost-effectiveness analysis which includes health outcomes in the analysis (such as quality adjusted life year (QALYs))	Carroll and Downs, (2006)
Budget Impact analysis (also called ‘business case analysis)	A type of economic assessment that estimates the financial consequences of adopting a new intervention and evaluates whether the high-value intervention is affordable. A process that provides the best-value analysis that considers not only cost but also other quantifiable and non-quantifiable factors supporting an investment decision	Garattini and van de Vooren, (2011)
Return of Investment	A way to calculate the financial gains (or losses), while taking into account all the resources invested and all the amounts gained through increased revenue, reduced costs, or both	Bertram et al. (2018); Stenberg et al. (2016)
Social Return of investment	A pragmatic form of cost-benefit analysis that measures the social value generated by an intervention by considering its broader impact on all stakeholders within the locality of the intervention and incorporating social value where it is appropriate	Banke-Thomas et al. (2015)

**TABLE 3:** Selected models proposed to address NBS expansion*.

Type of Model	Description	References
Decision analytic model	A framework for compiling clinical and economic evidence in a systematic fashion, determining your product’s value, and communicating that value to decision makers.	Grosse et al. (2016); Prosser et al. (2013), Prosser et al. (2018) www.treeage.com
Markov Model	A mathematical model using the probabilities of different health states and the rates of transitions among them to recognize patterns, make predictions and to apply the statistics of sequential data.	Chan et al. (2011) www.treeage.com
Discrete Event Simulation Model	A method of simulating the behavior and performance of a real-life process, facility, or system.	Salleh et al. (2017) www.mathworks.com
Microsimulation model	A method of using individual-based state-transition models to reflect individual clinical pathways, incorporate the impact of history on future events, and capture the variation in patients’ characteristics at baseline.	Verkleij et al. (2021) www.treeage.com
Agent-based model	A computational model for simulating the actions and interactions of autonomous agents in order to understand the behavior of a system and what governs its outcomes.	Tracy et al. (2018) www.anylogic.com
System dynamic models	A computer-aided approach for strategy and policy design, which can portray processes of accumulation and feedback and that may be tested systematically to find effective policies for overcoming policy resistance.	Yu et al. (2019) https://systemdynamics.org
System thinking models	A way of approaching problems that asks how various elements within a system, (which could be an ecosystem, an organization, or something more dispersed such as a supply chain) can influence one another.	Carey et al. (2015) www.vensim.com

* Table 3 highlights the different models that can be used to conduct the different analyses indicated in Table 2. The availability of models is not limited to the list depicted here.

## What are models?

Models can be used to simulate a reasonable representation of real-life scenarios. In NBS expansion, decision modeling can be used to study the *“context*” and *“complexity”* of a condition that is a candidate for a NBS pilot or for the RUSP. Models can inform how screening for a condition may transpire during state-wide implementation and/or adoption. By *context*, we can define the study population, the natural history of the disease, and the treatments, interventions, and management approaches that are to be studied. Context can also help decision-makers determine the portion of the problem to be included in the analysis. For example, in the case of conducting a cost-effectiveness analysis for lysosomal storage disorders (LSDs), we may ask ourselves, “Do we consider the consequence (cost and benefit) of the detection of possible comorbidities (i.e., deafness, blindness, pulmonary, and cardiac problems) in our decision making?” By *complexity*, we can define the appropriate scope and parameters of the NBS system component(s) to include in the analysis. The complexity of the analysis will depend on the study’s purpose, the availability of data, and the time allotted for the study’s design and examination. The *time horizon* of the model describes the study’s length of time, which can be informed by the length of a typical research pilot. The model can also include the time frame of the natural history of the disease and the disease process and compare newborns identified through NBS versus clinical presentation of symptoms. The findings from modeling could be a part of nomination information submitted to the ACHDNC.

The ACHDNC reviews the nominations to the RUSP and the evidence review process defines the net benefit of early identification through NBS and quantifies the opportunity for early treatment as compared to identification through symptomatology and clinical presentation and presumably later treatment. For example, a decision analysis model for NBS screening for spinal muscular atrophy (SMA) can be used to examine the time horizon of six-months with early treatment after NBS identification, compared to later treatment in the absence of NBS SMA screening. As another example: if the goal is to understand to the long-term benefits of treatment administrated at six-month over the next 5 years, a Markov model can be used to understand the long-term benefits of early treatment by modeling a 5-years time horizon comparing health outcomes resulting from interventions that occurred at different disease progression stages. Models can be applied prospectively throughout the pilot study as well as retrospectively after the pilot is completed.

## Potential use cases and models in newborn screening

While there are many models to select from, additional research is needed to determine which models work best, to develop additional models if needed, and then to apply the model(s) to address NBS expansion challenges. In this paper, we highlight how one “could” use models to facilitate NBS expansion in the United States. For each factor listed in Table 1, we describe 1) the *“use case,”* which highlights how the identified factor has delayed NBS expansion, 2) the “potential solution(s)” in addressing the challenges, and 3) a “*model*” that could be developed and applied to solve or address the challenges. The model(s) suggested below is an example for discussion; thus, we believe additional research is needed to support the development of models to further the discovery of solutions in addressing the challenges.

### Factor 1. Variability in screening panels persists

a. Use Case: State NBS screening panels shows that the number of conditions screened ranges from a low of 32 core conditions to a high of 71 core, secondary, and non-RUSP conditions combined, which indicates the persistence of variability in the composition of NBS screening panels by state. A total of 81 different conditions are screened across the United States Addition of conditions to screening panels is done by individual states, and each develops its own screening and follow-up algorithm. ACHDNC has established a nomination and evidence review process that established the RUSP. However, state laws can mandate screening for conditions not included on the RUSP. These state-specific legislative mandates and differences in practices lead to implementation differences across the United States and limit opportunities to systematically apply best practices, assess quality, and aggregate data. The lack of systematic data collection and interoperability between states makes it challenging to obtain and maintain data regarding barriers and facilitators of screening for new conditions within NBS.
b. Potential Solutions(s): To help decrease the variability in screening panels, states could adopt a real-time tracking and assessment of state practices that is assessable on a shared platform (such as on NBSTRN).
c. Models: With modeling, the researchers can use real-time data (the number of cases identified) and assumptions (the different treatment options—conventional treatment vs experimental treatment) to simulate different scenarios (to screen with test 1 vs test 2) to test different hypotheses (to screen 50,000 vs 100,000 babies per year in the pilot study). For example: It took 10 years for every state to implement screening for severe combined immunodeficiency (SCID). During the 10 years, a baby born with SCID in a state that was not offering SCID screening may not had been identified early enough to benefit from treatment before the onset of opportunistic infections. In some cases, SCID babies identified through the presentation of clinical symptoms did not survive and/or had a more challenging course of treatment and poorer outcomes. Data collected at the state level could be used to document the variation in practices and be used to inform models to explore the impact of screening vs not screening as well as the impact of different screening approaches. This has the potential to provide guidance to policymakers and decision-makers to support implementation in their own state based on the state-specific contextual factors included in the model. A decision analytic model could be used to document the increase in number of individuals who achieve the best health outcomes when all states adhere to a uniform screening panel. The decision analytic model could be used to define cost-effectiveness (comparing screening vs no-screening), cost-utility (examining the quality adjusted of life) with screening, or cost-of-illness analysis (to account for the additional inpatient hospitalization and other health expenditures related to care of the patient identified with a late diagnosis of a genetic condition). The model uses different cost ranges for the NBS screening components (point of care or laboratory equipment, reagents, expertise, quality assurance (QA) etc.). To project the budget to offer screening for a specific new condition or to use a new screening instrument for a current RUSP condition, a business case analysis template could be developed for different states with varied population sizes to account for operationalization factors (such as hospital versus laboratory screening, the salary of NBS team members), effort, contractual issues, upgrading, and maintenance support for implementing screening. Creating a system of models (or templates) used for projecting cost and benefits can help facilitate the adoption and implementation of new conditions.

### Factor 2. The short duration of pilots limits information about interventions and health outcomes

a. Use Case: There are no standardized protocols used to conduct NBS research, implementation, and/or adoption pilots. NBS research pilots often end when a single newborn with the targeted condition has been identified, and the diagnosis confirmed. In contrast, implementation pilots may screen for a pre-determined duration or until ∼80,000 newborns have been screened. Research and implementation pilots usually provide sufficient data to determine the analytical and clinical validation of at least one state-specific screening test and algorithm. For a state to expand its panel to include a new condition, an adoption pilot that replicates the analytical and clinical validation studies of the research and/or implementation pilots is required, and the results are not typically published. The amount of funding that is available to support NBS pilots as well as their short timeframe does not support the longitudinal data collection that is necessary to assess the benefit of early identification through screening, including information about the type, duration, and availability of treatments and the health outcomes of treated individuals. However, models can be used to simulate the natural history and clinical course of a patient identified through NBS beyond the pilot study duration.

#### DMD pilot study as a case study

NBS for Duchenne Muscular Dystrophy (DMD) is a useful case study for several reasons: 1) DMD is relatively common with ∼1 in 5,000 males diagnosed with DMD, 2) X-linked inheritance leading to carrier identification in mother’s and other family members, 3) an FDA-cleared kit for NBS is available, 4) two advocacy groups operate longitudinal patient registries that provide health outcome data, 5) the presentation of clinical symptoms and average diagnosis of over 4 years of age often results in a second, younger child in the family having DMD which helps sets up an informative comparison in early versus later treatment, and 6) new treatment and management approaches provide “before and after” scenarios that are useful for comparisons.

b. Possible Solution(s): Research is needed to understand the health services and medical management of positively screened individual beyond the NBS pilot study duration. The findings can help create an infrastructure of long-term follow-up that includes care coordination and data collection to inform clinicians, state programs, and families with the goal of improving the care and needs of the affected individuals.
c. Models: While the medical and health data for the affected individuals may be limited, using models such as decision analytic models, Markov models, and/or system dynamics models can simulate different health pathways and the impact of different interventions in hypothetical settings. These models can test a range of variables that may be sensitive to the NBS expansion process and nationwide adoption including: 1) population size, 2) duration of the pilot, 3) incidence rate, and 4) workforce capacity at the state based NBS program (www.vensim.com). Decision analytical models use parameters such as an incidence rate, specificity, and sensitivity of the screening test, as well as the positive predictive value to project the effectiveness of screening. Thus, it is possible to use models as needed to identify a specific number of cases with a genetic condition that could be expected in a given population size. For DMD, an incidence of 1 in 5,000 means that one could expect to identify a newborn with DMD in the first 5,000 newborns screened. The use of patient registries to compare outcomes of the affected members in families with more than one child is a useful surrogate for long-term follow-up outcome studies. In fact, once a family history of DMD is documented and/or a mother is identified as a carrier of DMD, prenatal identification of DMD could mimic NBS identified DMD and help add data to determine whether early identification, management, and treatment improves outcomes. In addition, policy makers want to assess the impact of adding a screening test. To understand the impact of making a change to the system, a system dynamic model that studies the impact of “feedback loops” into the system could be used. Feedback loops are used to capture the interactions between the parts of the system and how they lead to a certain overall pattern of trend over time and are described as a *positive feedback loop* or *negative feedback loop*. For example, a screening test with a higher sensitivity may result in an increase in positive cases which is an example of *positive feedback*, while screening test with a lower specificity may result in an increase in false positives which is an example of *negative feedback*. The increase in false positives may then lead to an increase in parental anxiety due to unnecessary follow-up testing and increase health care costs (another example of *positive feedback*). System dynamic models can help identify areas in the system where changes to policy (i.e., improving specificity rate to reduce false positives) will have the highest return on investment. ACHDNC uses NBS pilot data to determine whether to recommend addition of a condition to the RUSP. NBS pilots of short duration may provide sufficient data if there is surrogate data for outcomes such as patient registries, families with multiple affected individuals and/or prenatal identification.

### Factor 3. Recent additions to the recommended uniform screening panel (RUSP) expand the definition of newborn screening

a. Use Case: Several hallmarks of NBS are evolving based on recent additions to the RUSP, including age of disease onset and the need for neonatal treatment. In addition, past efforts have shown that once a condition is screened on a population basis a spectrum of clinical disease, beyond the target condition, is often discovered (Puck, 2019). While there are some diseases with a strong correlation between genotype and age of onset (e.g. multiple endocrine neoplasia, type IIB)*,* the current RUSP is organized into core and secondary conditions with variable onsets and/or defined late-onset forms that will manifest far beyond the newborn period, if at all. The fine line between individuals who will be late onset versus non-penetrant complicates diagnosis as well as decision-making regarding when and whether to treat.

#### Case example infantile vs. late-onset Pompe disease

Pompe disease has both infantile (IOPD) and late-onset (LOPD) forms. Newborns with IOPD have muscle problems that begin in early infancy and these problems can worsen quickly and cause death within the first year. Most newborns who have a positive NBS screen have LOPD, thus symptoms may not appear until later childhood throughout adulthood. This means that a condition identified through NBS may not be actionable until adulthood, if at all (https://www.hrsa.gov/sites/default/files/hrsa/advisory-committees/heritable-disorders/rusp/previous-nominations/pompe-27-june-2018.pdf).

b. Possible Solution(s): To capture a diverse of perspectives on the addition of condition on the RUSP, an ongoing real-time survey collecting information regarding facilitators and barriers of NBS expansion could be created. Also, these insights can be extrapolated from stakeholders on a NBSTRN Forum which is a secured site for a member directed-discussion board. A best practices checklist for diagnosis, intervention, and management can be generated from this community of diverse stakeholders. For example, from these discussion board insights or real-time survey, a *short-term* follow-up data can be used in models to project *long-term* health outcomes.
c. Models: A Markov model can be created to simulate health states beyond the newborn period and project different health outcomes scenarios. In the case of IOPD vs LOPD, Markov models describing affected individuals identified by NBS for IOPD can be compared to LOPD, and used to understand the impact of different diagnostic, treatment, and management approaches beyond the NBS pilot study.

### Factor 4. The RUSP nomination and evidence review process has capacity constraints

a. Use Case: ACHDNC mostly reviews one condition at a time and in some special cases, two conditions. State NBS program readiness for expanded screening is not standardized because many factors impact the implementation process. Different entities fund different aspects of the various pilot studies, and there is a lack of coordination and alignment of pilot goals (refer to companion Paper One) (Brower et al., 2022). There is a lack of information about the development and measurement of economic outcomes from both the National and State Program perspectives. This is exemplified by the scenario where one State program covers all cost of care for a condition if managed through State metabolic program, whereas another State program plays no role in the care coordination. One challenge is the lack of direct assessment of the impact of NBS expansion on health care providers including primary care, specialty physicians, genetic counselors, and other allied professionals. In addition, aspects related to funding varies across states and may change on a monthly or annual basis. Because the goal of NBS is to improve health outcomes through early diagnosis and treatment, an assessment of the benefit of NBS requires longitudinal health information. Although longitudinal data collection may be possible, there is no national registry or system to collect this information, and the complexity of some NBS conditions makes the determination of the clinical relevance more challenging such as specific disease issues related to milder expression, novel forms of disorder identified through screening, later onset, non-penetrance, carriers, and X-linked. Data sources are also diverse and not always easily accessible (such as school data). The majority of NBS conditions require lifelong treatment and management, therefore health outcomes may take years or decades to accumulate, and NBS pilots are not designed to meet this need.
b. Possible Solution(s): NBS expansion most often occurs one condition at a time and is triggered either by the nomination, evidence review and recommendation on a national level by the ACHDNC or by the adoption of new state laws. A solution could be an overarching system that collects data over time of NBS conditions in pilot studies or simulated pilot studies which could provide an evidence base, identifies, and archives the parameters that support the implementation of multiple candidate conditions simultaneously. For example, if a set of conditions have similar expected incidence rates and the screening tests have acceptable specificity and sensitivity rates, then the use of a model can shorten the duration of a pilot studies which are focused on the analytical and clinical validation of the screening tests to a few months (instead of 18 months). The model can also be used to simulate and extend the duration of pilot studies as needed. This data system could also include longitudinal health records that could be made available to parents and caregivers to improve communication with the healthcare team and lessen care disparities. With input from multiple stakeholders from different state, this could facilitate a “regional state” approach for adoption, and even screening, instead of the current approach (state by state); with input about multiple conditions, we could facilitate the adoption of more than one condition. Real-time models can be created to simulate input from parents and physicians on the different late-onset disorders using a unified database providing similar data and data fields such as the NBSTRN Longitudinal Pediatric Data Resource (LPDR). We can also explore a collaborative model with industry for new experimental diagnostic and treatment technologies for new conditions, clinical care for new interventions, treatments and management approaches, and state based NBS programs to identify new cases and coordinate timely referral and care.
c. Models: These types of simulation models have been used to project health outcomes. For example, estimates of the number of lives in a large population saved from infections due to vaccination and documentation of the subsequent reduction in disease incidence, uses preliminary data obtained from smaller populations. Further research is needed to determine which models can be used to best predict the impact of using different approaches for adoption (regional versus individual state; more than one condition versus one condition). It is also important to note that the ability to model the proposed scenarios would be predicated on the sharing of data via a repository or some other such infrastructure in a concerted effort to facilitate such an effort. NBSTRN created the data tool, LPDR to support an infrastructure for data sharing for secondary use of the original data set. The data extrapolated from these data set would an example of secondary use for modelling.

## Discussion

As the number and type of conditions that would benefit from early identification and intervention through NBS continues to increase, models can be employed to rethink and reimagine the process that traditionally governs NBS expansion and the approach to pilot studies from research to state and nationwide adoption can be improved. NBSTRN has created an array of data tools (LPDR, NBS-CR, NBS-VR, and ELSI Advantage) to facilitate secondary use of original data sets because the ability to capture clinical information early in the clinical course of a disease can help advance our understanding of disease etiology, contribute to new knowledge for new treatments and therapy development, and identify areas for improvement in disease management throughout the lifespan for affected families (https://nbstrn.org/tools). The use of modelling can help further address the challenges described in *Population-based Screening of Newborns: Findings from the NBS Expansion Study (Part One)* (Brower et al., 2022).

## Advantages of using models

The advantages of using models include: 1) reducing expense in comparison with conducting a large-scale pilot study; 2) estimating the public health and clinical outcomes from models is timesaving compared to the time horizon of a typical pilot study (i.e., at least 1–2 years); 3) simulating different real-world scenarios (i.e., different cut-off levels); and 4) informing the design of a pilot study and identifying those outcomes most critical to measure in the pilot. Models depend heavily on data inputs, and the quality of the data will impact the robustness and validity of the model outcomes. Several options can be considered to inform the data inputs including real-world data from prior implementations, robust data-informed assumptions, and the use of expert opinions for reasonable estimates when data are not available, coupled with sensitivity analyses described below.

One of the concerns for using models is the uncertainty or variation in the model assumptions, which can significantly impact the outcome. To address uncertainty, sensitivity analysis is a powerful tool that explores the variability of the model under different sets of assumptions, including different incidences, different population sizes, and different cut-off levels based on specificity and sensitivity screening parameters. Decision modeling and sensitivity analysis can accompany small-scale pilot studies to determine the which inputs are most “*sensitive*” to variation and assess how this may impact conclusions, decision-making, and screening policies. For example, a policymaker may be deciding whether to allocate funding to support implementation of a state-wide screening program for a new condition, and while the true incidence of the condition is unknown, the reported range is between 1 in 25,000 and 1 in 500,000. A model coupled with a sensitivity analysis studying model outputs based on incidence rates between 1 in 25,000 and 1 in 500,000 could determine the incidence threshold at which the program would be deemed cost-effective (a threshold analysis). The probability that the incidence falls at or above the threshold can be determined by the model.

## Future directions using models for NBS expansion

To help guide NBS expansion and create a roadmap for improvement, NBSTRN is like the “hub” of the wheel, where diverse stakeholders such as clinicians, researchers, state NBS programs, families, and advocacy organizations are among the “spokes”, driving implementation and innovation. To realize the promise of models for NBS expansion, new stakeholders from system sciences, health economics, supply chain management, data engineering, and communication must be additional spokes of the wheel. Artificial intelligence and machine learning have also been used on existing screening data to improve the prediction of true and false positive results (Peng et al., 2020), and this is an additional area of interest as we work to identify new strategies. The development of interdisciplinary efforts and systems approaches to implementation could help advance NBS research and improve NBS expansion.

An ideal scenario is for researchers conducting NBS pilot studies to partner with system scientists to develop models that simulate and project the consequences of expanding NBS by exploring different model parameters. NBSTRN is a designed to facilitate these types of innovative efforts in newborn screening-related research to discover new screening technologies, treatments, and interventions. As a key component of the *Eunice Kennedy Shriver* NICHD Hunter Kelly NBS Research Program, NBSTRN can continue organizing network meetings to bring together the different disciplines to create models to evaluate the different NBS scenarios. ACMG has developed and coordinated the NBSTRN since its beginning in 2008, and the alignment between NBSTRN objectives and ACMG’s mission enhances the NBSTRN ability to advocate for improvements in NBS. For example, instead of carrying out an 18-months pilot study traditionally, State Department of Public Health can collaborate with system scientists to use data in real-time to project the likelihood of identifying a case and if case is identified, what is the likelihood of obtaining treatment early to yield a ‘better’ health outcome (improved quality of life for the baby with the condition and family). In conjunction with tools and specialized training provided by the NBSTRN, models can be used to evaluate the impact of barriers (i.e., lack of infrastructure) and facilitators (i.e., sufficient funding) in NBS pilot studies. To support the use of models in a pilot study, additional funding is needed to support modeling research and implementation to hypothesize whether models could be used, and if used, under what conditions, parameters, and assumptions. With appropriate funding to support online training and in-person workshops on the fundamentals, application, and implementation of models, this new innovative new approach can be broadly used for conducting pilot studies as well as for policymaking. Thus, to address these needs and foster collaboration for new solutions using modeling, active and growing membership of diverse expertise in and support of the NBSTRN network is critical for developing new approaches to advance and sustain NBS research.
